# Human Parainfluenza Virus-Associated Respiratory Tract Infection among Children and Genetic Analysis of HPIV-3 Strains in Beijing, China

**DOI:** 10.1371/journal.pone.0043893

**Published:** 2012-08-28

**Authors:** Naiying Mao, Yixin Ji, Zhengde Xie, Huanhuan Wang, Huiling Wang, Junjing An, Xinxin Zhang, Yan Zhang, Zhen Zhu, Aili Cui, Songtao Xu, Kunling Shen, Chunyan Liu, Weizhong Yang, Wenbo Xu

**Affiliations:** 1 World Health Organization Regional Office for the Western Pacific Regional Reference Measles Lab and State Key Laboratory for Molecular Virology and Genetic Engineering, National Institute for Viral Disease Control and Prevention, Chinese Center for Disease Control and Prevention, Beijing, China; 2 Beijing Children’s Hospital, Capital Medical University, Beijing, China; 3 Department of Neurobiology, Taishan Medical College, Taian, Shandong, China; 4 Office for Disease Control and Emergency Response, Chinese Center for Disease Control and Prevention, Beijing, China; Centers for Disease Control and Prevention, United States of America

## Abstract

The relevance of human parainfluenza viruses (HPIVs) to the epidemiology of acute respiratory infections (ARI) in China is unclear. From May 2008 to September 2010, 443 nasopharyngeal aspirates (NPAs) from hospitalized pediatric patients (age from 1 to 93 months) in Beijing were collected and screened for HPIVs and other common respiratory viruses by real-time RT-PCR. Sixty-two of 443 samples were positive for HPIVs with 4 positive for HPIV-2 and 58 positive for HPIV-3, indicating that HPIV-3 was the predominant virus present during the study period. A phylogenetic tree based on all the available HN (hemagglutinin-neuraminidase) sequences of HPIV-3 indicated that three distinct clusters (A,B, and C) were circulating with some temporal and regional clustering. Cluster C was further divided into sub-clusters, C1, C2, C3 and C4. HPIV-3 from Beijing isolates belonged to sub-cluster C3, and were grouped with the isolates from two Provinces of China and the neighboring country of Japan. Genetic analysis based on entire HN gene revealed that the HPIV-3 isolates from Beijing were highly similar with 97.2%–100% identity at the nucleotide level and these could be divided into two closely related lineages, C3a and C3b. These findings suggested that there was co-circulation of multiple lineages of HPIV-3 in the Beijing region during the study period. This is the first study to describe the epidemiology and molecular characterization of HPIVs in China.

## Introduction

Acute respiratory infections (ARI) are associated with significant morbidity, especially among infants and young children [1], [2]. One estimate suggested that pneumonia accounted for 19% of the 10.6 million yearly deaths in children younger than 5 years in 2000–03, and was the leading cause of childhood mortality among this age group globally [3]. Human parainfluenza viruses (HPIVs) are not only a common causative agent of ARI among infants and young children, but these viruses are also associated with nosocomial acute respiratory illness in the immunocompromised, hematopoietic stem cell transplant patients [4], [5], [6]. In the USA, it is estimated that 7600 to 48000 among children age <1 year old and 8100 to 42600 children age 1 to 4 years were hospitalized with HPIVs infection annually [7].

HPIVs are enveloped, single-stranded negative sense RNA viruses that belong to the Family *Paramyxoviridae* [8]. Based on genetic and antigenic variation, HPIVs have been divided into types 1 to 4 [9]. The majority of their structural and biological characteristics are similar, but each type infects human at different ages and causes different diseases, such as upper respiratory infection, croup, bronchitis, and pneumonia [10], [11]. Almost all children encounter HPIVs within the first few years after birth, but immunity is incomplete and re-infections occur throughout life [12]. HPIV-1 is the most common pathogen associated with croup and is marked by biennial fall epidemics [13]. The majority of HPIV-1 infections occur in children aged 7 to 36 months [12], [14]. HPIV-3 infections occur yearly, mainly in spring and summer [15].Unlike other HPIVs, approximately two-thirds of children are infected by HPIV-3 in the first year of life, mainly causing bronchiolitis and pneumonia [15]. Outbreaks of HPIV-2 usually follow HPIV-1 outbreaks depending on the time of years [16], [17], [18]. The majority of HPIV-2 infections occur in children younger than 5 years of age [6].

The viral envelope of HPIVs contains two major surface glycoproteins: the hemagglutinin-neuraminidase (HN) and the fusion (F) proteins [19]. The HN glycoprotein regulating the interaction between virus and host cells, has dual biological functions of hemagglutinin and neuraminidase activities and also plays an essential role in promoting fusion by the F protein [8]. In addition, the HN glycoprotein possesses the largest antigenic and genetic differences among HPIV types and strains within one type [20], [21], [22]. Therefore, the HN glycoprotein, which is the target for protective humoral immunity, has been broadly used for typing HPIVs for molecular epidemiological investigations [23], [24], [25], [26].

In China, the incidence of HPIV infection is underestimated. Its epidemiology is poorly understood due to the difficulties with diagnosis in most clinical virology laboratories. Fewer studies have been conducted on sequence variations in HPIV-3, and most were based on sequencing of only limited regions of the HN glycoprotein [27]. In the present study, 443 hospitalized children with ARI over 29 consecutive months were screened by real-time RT-PCR for respiratory viruses, including HPIV1-3, human respiratory syncytial virus (RSV), human rhinovirus (HRVs), adenovirus (AdV), human coronaviruses(HCoV) and human metapneumovirus (HMPV). In order to investigate the genetic characteristics of HPIV-3 that was circulating in the Beijing region, the complete HN gene was amplified from those specimens positive by HPIV-3 real-time RT-PCR and sequenced. Clinical information was collected from all patients.

## Results

### Patient Characteristics

From May 2008 to September 2010, 443 nasopharyngeal aspirates were collected from approximately 2500 patients with ARI who had been admitted to Beijing Children’s hospital. During the 29-months study period, 273 (61.6%) patients from May 2008 to Apr 2009 (2008–2009 season), 136 (30.7%) patients from May 2009 to Apr 2010 (2009–2010 season), and 34 (7.7%) patients from May 2010 to Sep 2010 (2010 season) were enrolled. The hospitalized children with ARI aged from 0 to 93 months among which 66.1% (293/443) were younger than 12 months. The median age was 14 months. The ratio of boys to girls was 1.7∶1. Among the enrolled children, pneumonia was the most common clinical diagnosis (376, 84.9%), followed by bronchitis (30, 6.8%), bronchiolitis (21, 4.7%) and upper respiratory tract infections (16, 3.6%).

### Detection of HPIVs and Other Respiratory Virus

Out of the 443 specimens, at least one respiratory virus was detected in 366 specimens (83.6%). The most frequently detected virus was RSV(169, 46.2% of positive patients), followed by HCoV (134, 36.6%), HRV (123, 33.6%), HMPV (66, 18%) and HPIVs (62, 16.9%). HIPV-3 accounted for 15.8% (58/366) of the total viral agents and was the fifth most frequently detected viral pathogen in our study. HPIV-2 was detected in 4 cases during the study period. Additionally, 76% (44/58) of all HPIV-3 positive children were found to be coinfected with other respiratory viruses, including 28 with 1 respiratory virus, 14 with 2 respiratory viruses, 1 with 3 respiratory viruses, 1 with 4 respiratory viruses (Table 1). All HPIV-2 positive children were found to be coinfected with other respiratory viruses including 1 with RSV, HCoV, HMPV and HRV respectively. HPIV-1 was not detected in our population during the study period.

**Table 1:** Co-infections in HPIV-3 positive Samples.

No. of Cases	Respiratory viruses detected	Diagnosis
15	HPIV-3, HCoV	15 pneumonia
5	HPIV-3, HRV	5 pneumonia
3	HPIV-3, RSV	2 pneumonia, 1 bronchitis
3	HPIV-3, HMPV	2 pneumonia, 1 bronchitis
2	HPIV-3, ADV	2 pneumonia
6	HPIV-3, HCoV, HRV	5 pneumonia, 1 bronchitis
1	HPIV-3, HCoV, HMPV	1 pneumonia
1	HPIV-3, HCoV, ADV	1 pneumonia
1	HPIV-3, HCoV, RSV	1 pneumonia
2	HPIV-3, HMPV, RSV	1 pneumonia, 1 bronchiolitis
1	HPIV-3, ADV, RSV	1 pneumonia,
1	HPIV-3, RSV, HRV	1 pneumonia
1	HPIV-3, ADV, HRV	1 pneumonia,
1	HPIV-3, HCoV, HMPV, HRV	1 pneumonia,
1	HPIV-3,ADV, RSV, HRV, HMPV	1 pneumonia,

### Epidemiology of HPIVs

**Figure 1:**
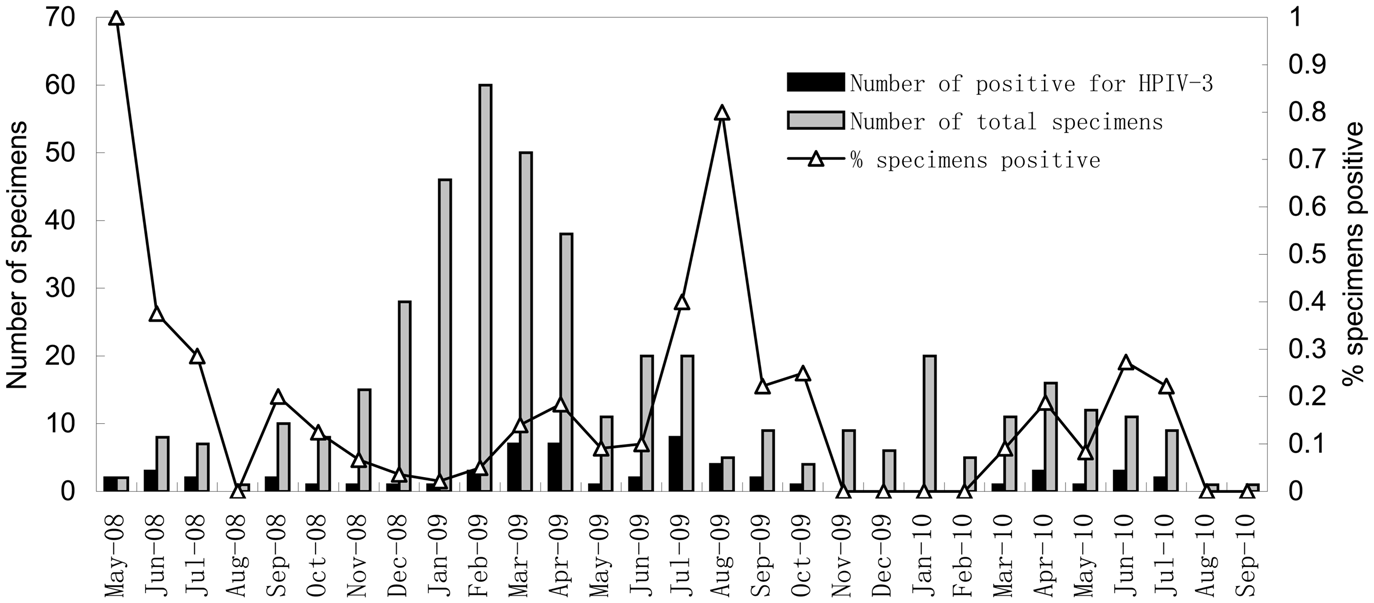
Monthly distribution of the 58 hospitalized children with HPIV-3 infection.

HPIV-3 was detected throughout the study period and the positive rates of HPIV-3 in spring, summer, autumn and winter were 16% (22/140), 29% (24/82), 13% (7/56) and 3% (5/156) (Figure 1). The majority of the HPIV-3 infections (79%, 46/58) occurred in spring and summer (March to August). The age of HPIV-3 infected patients ranged from 0 to 26 months. There was a significant difference observed in the relative prevalence of HPIV-3 positive patients younger than 12 months(15.7%, 46/293) compared to children older than 12 months (8%, 12/150) of age (Chi-square test: p = 0.023). HPIV-3 infected patients were significantly younger than respiratory viruses negative patients (median of 6 versus 18 months) (Mann-Whitney U test; P = 0.000). The male/female ratio in the HPIV-3 infected group was 2.2∶1. In contrast, in the HPIV-3 negative group, the ratio was 1.6∶1, although this difference was not significant (chi-square test: p = 0.277). HPIV-2 was only detected in December 2008, May 2009 and January 2010 within children under 16 months old.

### Genetic and Amino Acid Analysis of HPIV-3

The complete HN sequences (1719 nt in length) of 42 HPIV-3 isolates from 58 HPIV-3 positive specimens, which were collected from May 2008 to October 2009, were analyzed. The HN gene failed to amplify from 16 HPIV-3 positive specimens, which had C_t_ values ranging from 29 to 31 in real-time RT-PCR reaction. The failure to amplify could be due to the mismatches in the primer binding sites as a result of genetic diversity among strains of HPIV-3. The nucleotide sequence and deduced amino acid homologies among these Beijing HPIV-3 isolates were between 97.2 and 100% and 98.7 and 100%, respectively. All genetic changes in the HPIV-3 isolates evaluated in this study were base substitutions without deletions, insertions, or frame-shift mutations. The mutations found among the different isolates were scattered throughout the HN gene. The divergence between the Beijing isolates and the prototype strain Wash/47885/57 was approximately 4.6–5.2% and 2.3–3% in the nucleotide and deduced amino acid sequence, respectively. Compared to the prototype strain, the Beijing HPIV-3 isolates shared 11 unique amino acid changes, Met21Thr, IIe40Thr, His62Arg, Lys63Glu, IIe76Val, Met118IIe, IIe143Thr, Ser318Leu, Val348Ala, IIe391Val, and Ser555Leu.

**Figure 2:**
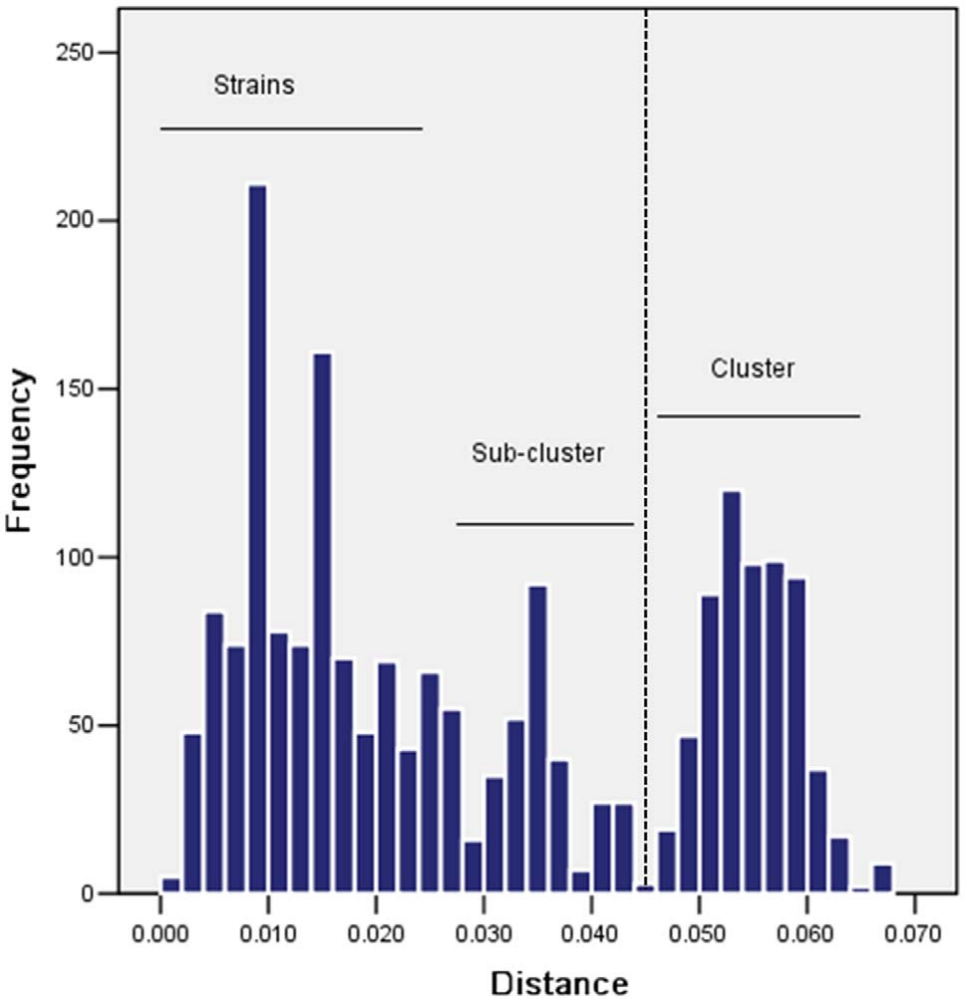
Genetic distances among HPIV-3 isolates. Nucleotide sequence diversity was calculated using Kimura 2-parameter method. The threshold value of 0.045 that divided comparisons between members of the same (phylogenetically defined) cluster and between different clusters is shown as a dashed line.

Pairwise distances between the sequences were calculated using the Kimura 2-parameter method. The numbers in the distance matrix (data not shown) were plotted into a histogram that formed three peaks corresponding to the distance ranges suggesting the presence of strains, sub-clusters and clusters, respectively (Figure 2). Two distance ranges did not overlap suggesting the HPIV-3 can be unambiguously classified into two distinct categories: strains and cluster. However, a minor peak occurred between strain and cluster range, indicating the diversity of stains within cluster.

### Phylogenetic Analysis of HPIV-3

**Figure 3:**
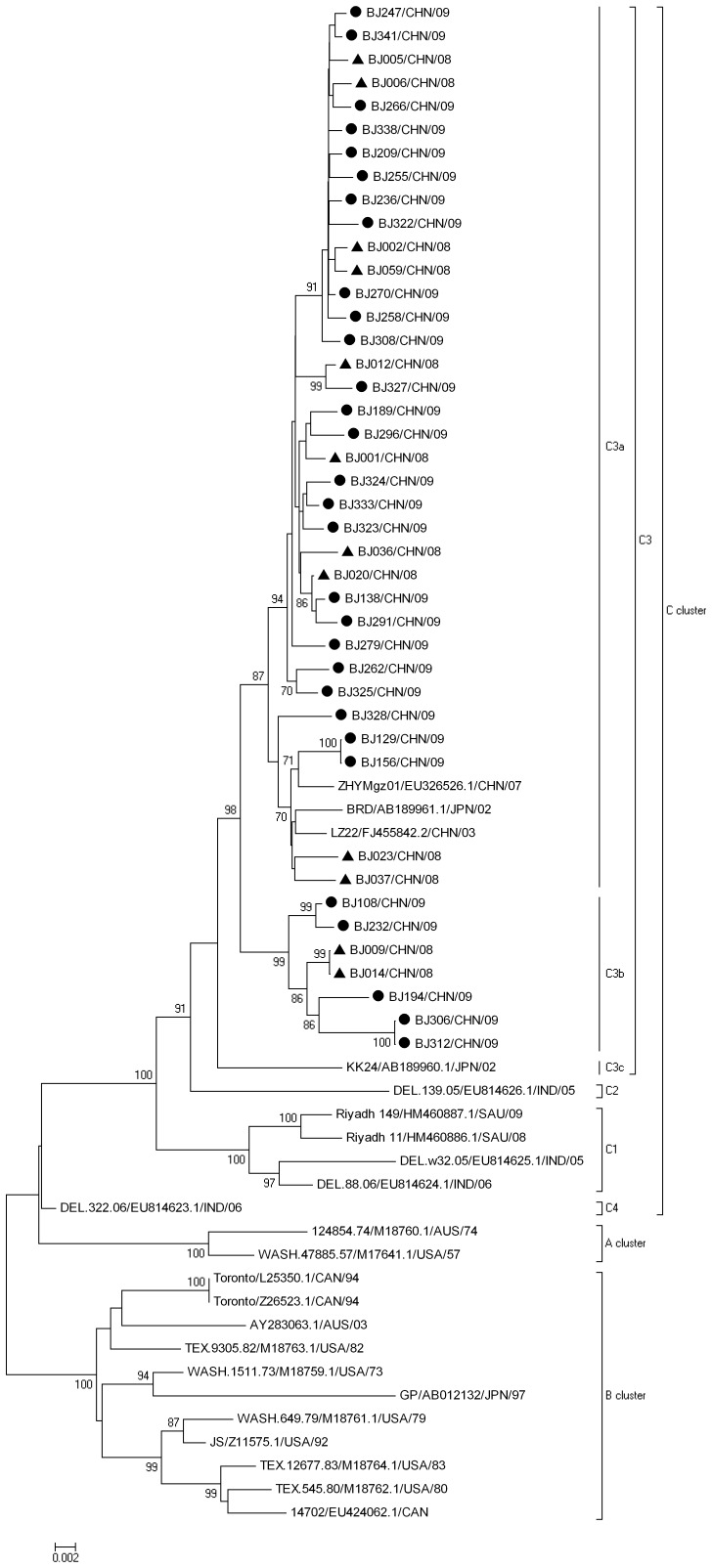
Phylogenetic analysis based on complete HN sequences of Beijing strains and other strains of HPIV-3 from different geographic origins. The neighbor-joining method was used to construct the trees. Numbers at the nodes represent the percentage of 1,000 bootstrap pseudoreplicates.▴2008• 2009.

In the present study, the HN sequences from all of the Beijing HPIV-3 isolates obtained were aligned with the sequences of 23 HPIV-3 isolates from 7 countries (USA, Australia, India, Japan, Canada, Saudi Arabia and China), to construct the phylogenetic tree (Figure 3). The tree constructed by the Kimura 2-Parameter method is highly similar to that produced by Maximum Likelihood method (data not shown). Phylogenetic analysis showed that all 65 HPIV-3 isolates were divided into three distinct clusters (A, B and C) with at least 4.5% nucleotide divergence between clusters. Cluster A consisted of the prototype strain Wash/47885/57, and one isolate from Australia. Cluster B included 11 isolates from the USA, Japan, Australia, and Canada with a mean nucleotide divergence of 2.5%. Interestingly, most of the isolates from Cluster A and B were isolated in the middle of the last century. Cluster Ccontained 52 isolates which have been isolated in recent years from Asia and had a mean nucleotide divergence of 1.8%. All of the Beijing HPIV-3 isolates were in Cluster C and formed a monophylogenetic sub-cluster C3 with 2 isolates from Japan and 2 isolates from mainland China, with a mean nucleotide divergence of 1.7%.

**Table 2:** Human parainfluenza viruses real-time reverse-transcription polymerase chain reaction (RT-PCR) panel primer and probe sequences.

Assay, primer/probe	Final concentrationnmol/L	Gene target	Sequence(5′→3′)
HPIV-1^a^		HN protein	
Forward	900		GTTGTCAATGTCTTAATTCGTATCAATAATT
Reverse	900		GTAGCCTMCCTTCGGCACCTAA
Probe^c^	200		TAGGCCAAAGATTGTTGTCGAGACTATTCCAA
HPIV-2^a^		HN protein	
Forward	900		GCATTTCCAATCTTCAGGACTATGA
Reverse	900		ACCTCCTGGTATAGCAGTGACTGAAC
Probe^c^	200		CCATTTACCTAAGTGATGGAATCAATCGCAAA
HPIV-3^b^		HN protein	
Forward	600		GGAGCATTGTGTCATCTGTC
Reverse	600		TAGTGTGTAATGCAGCTCGT
Probe^c^	340		CGCGCTACCCAGTCATAACTTACTCAACAGC AACAGCGCG

a Primer and probe sequences for HPIV-1 and HPIV-2 are from F. Watzinger et al. [46].

b Primer and probe sequences for HPIV-3 are fromKate E. Templeton et al. [47].

c Labeled at the 5′ end with FAM and terminally quenched at the 3′ end with Black Hole Quencher-1.

Cluster C isolates were further grouped into four sub-clusters with bootstrap values of 91 to 100% (C1, C2, C3 and C4) (Figure 3) with the exception of lineage C4, which was supported by a lower bootstrap value. Forty-six sequences including the Beijing isolates were placed into the C3 sub-cluster and 4 isolates from India and Saudi Arabia were placed into the C1 sub-cluster. Sub-clusters C4 and C2 contained only a single isolate (DEL.322.06 isolate and DEL.139.05 respectively) from India. In addition, sub-cluster C3 lineages were designated C3a, C3b and C3c with at least 1.5% nucleotide divergence. In our study, 35 Beijing HPIV-3 isolates belonged to the C3a group together with the previously reported 2 isolates from Guangdong and Gansu provinces of China and 1 isolate from Japan. The remaining seven Beijing HPIV-3 isolates were placed into the C3b group. Both C3a and C3b strains have been co-circulating in the Beijing region during 3 epidemic seasons. The phylogenetic tree (Figure3) shows that some isolates from the same epidemic season in Beijing (BJ006/CHN/08 and BJ009/CHN/08; BJ322/CHN/09 and BJ237/CHN/09; BJ194/CHN/09 and BJ209/CHN/09; BJ306/CHN/09 and BJ308/CHN/09) can show a greater divergence than isolates from different sampling years (BJ006/CHN/08 and BJ266/CHN/09; BJ012/CHN/08 and BJ327/CHN/09). It is noteworthy that, compared with all cluster A and B, ten unique nucleotide substitutions in the HN coding region, T63C, G63A, G354A, A498G, G519A, G1233A, T1287C, T1314C, T1323C, G1464A, which caused 2 unique amino acid substitutions: Met21Thr, Met118IIe, were shared by all cluster C viruses.

## Discussion

In the present study, 443 specimens were collected from hospitalized children with ARI over 29 consecutive months and a significant rate of HPIVs infection (14%, 62/443) was observed, although, HPIVs were the fifth most frequent detected pathogen among the common respiratory-associated viral agents. This result demonstrated a substantial burden of HPIVs-associated ARI among hospitalized children which is consistent with previous studies in other countries, where HPIVs can be detected in 5 to 30% of hospitalized children with ARI depending on the year of study, case-definition and diagnostic technique used, the type of specimen collected, and seasonality [28], [29], [30], [31].

Even though HPIVs share common genetic and biochemical features, they differ in the age groups that they infect, seasonality, clinical manifestations. In this study, HPIV-3 was responsible for 94% (58/62) HPIVs infections, while HPIV-2 was responsible for only 4 cases and no HPIV-1 was detected. Most HPIV-3 infections occurred during a period of about 24 weeks during spring and summer. This result is consistent with previous studies, which showed that epidemics of HPIV-3 infection occurred in spring and summer [30], [32]. In addition, 53% (31/58) of HPIV-3 infected patients were younger than 6 months and 79% (46/58) of HPIV-3 infections were in the first year of life [9], [15].

A significant difference in the number of hospitalizations among HPIV-1, HPIV-2 and HPIV-3 infections detected by real-time RT-PCR was observed in this study. In general, HPIV-1 and HPIV-2 infection were important causes of lower respiratory infections and childhood hospitalization during the epidemic seasons [7]. Reports from the United States suggested that a minimum of 50% of croup cases were caused by HPIV-1 [10], [13], [33]. However, LRIs caused by HPIV-2 have been reported much lower frequencies than LRIs caused by HPIV-1 and HPIV-3 [7]. Our finding that HPIV-3 was the predominant HPIVs epidemic in the Beijing region may be because most specimens (84.9%) were collected from hospitalized patients with pneumonia whereas few specimens from bronchitis, bronchiolitis, upper respiratory infections and none from croup were tested. In addition, differences in the age distribution may be because younger children are at higher risk for HPIV-3 infections considering that most of patients (66.1%) enrolled in this study were younger than 12 months old. Similar findings reported in another study which showed that young infants (younger than 6 months) were particularly vulnerable to HPIV-3 infections [15]. However, long term studies are needed to determine the true prevalence of HPIVs in China.

Simultaneous infections by different viruses were observed in our study with 41.3% (183/443) of positive cases with mixed infections. This rate is significantly higher than those previously described in which co-infection rates ranged from 5 to 20% [34], [35], [36]. Interestingly, 77% (48/62) HPIVs infections were found as mixed infections. Among the HPIVs positive cases, co-infections with HCoV were most frequently found in children followed by HRVs, although RSV was the most common pathogen detected in our population. HRVs were identified in 16 out of 48 HPIVs co-infection cases, probably due to the increased detection sensitivity of real-time RT-PCR technologies. However, in the case of mixed infection, it has been hypothesized that picornaviruses could serve as a clinical illness promotion factor that functions additively or synergistically in the pathogenesis of lower respiratory syndromes such as bronchiolitis [37]. It was reported that RSV co-infection with HMPV may be a determination of disease severity [38], [39]. Therefore, further investigations are need to address high rate of co-infection with other respiratory viruses and to determine if the co-infections could results in enhanced severity of HPIVs.

Phylogenetic analysis revealed that all known HPIV-3 isolates could be divided into three distinct clusters (A, B and C) with at least 4.5% diversity between clusters. Cluster A and B contain strains mostly isolated from last century including the prototype strain, which seems to be less prevalent. Cluster C contains strains isolated from India, Japan, China and Saudi Arabia during 2003 to 2009, and was the predominant epidemic lineages of HPIV-3 in Asia. Cluster C was further classified into 4 sub-clusters C1–4. These findings indicate that cluster C isolates with distinct nucleotide divergence may spread widely. Although the Beijing isolates belonged to a single sub-cluster C3, multiple lineages of HPIV-3 co-circulating during the same epidemic season has been observed in this study. Lineage C3a, which contained 35 Beijing isolates, appeared to be more prevalent during the study period. Genetic analysis showed that HPIV-3 isolates were highly conserved with at least 95% and 97% identity at the nucleotide and deduced amino acid levels compared with the prototype strain (Wash/47885/57) which is consistent with previous reports [40]. Interestingly, 3 isolates, two of which are from distant provinces of China (Guangdong and Gansu) and one from the neighboring county of Japan, were genetically closely related to Beijing isolates and were grouped into lineage C3a. Our results implied that certain HPIV-3 strains have the potential to spread over different countries.

The epidemiology and the possible mechanisms by which HPIV-3 can evade the immune response are still need to be elucidated. Previous investigations suggested that the HPIV-3 F and HN glycoprotein have undergone little variation, with an overall low rate of noncumulative change and genetic heterogeneity [9], [41]. However, there has been conflicting evidence about whether the evolution of HPIV-3 is related temporally or geographically [42]. Sequence analysis of HPIV-1 and HPIV-3 strains identified that geographically-defined genetic lineages might develop [26], [43]. As shown in the phylogenetic tree, the isolates from China and Japan formed a unique branch in sub-cluster C3a. Most of the isolates from same country in recent years were likely to be clustered into the same lineages. These results suggested that variation within the HN gene of HPIV-3 could somehow be correlated with the geographic origin of the strain. Interestingly, ten unique nucleotide differences which cause 2 amino acid changes in cluster C were identified and seem to be accumulating mutations. It is in contrast to the pervious study in which the pattern of HPIV-3 was similar to that of influenza C virus with a non-progressive random variation [44]. However, because of absence of HPIV-3 sequences collected over time and that from other countries could limit our ability clarify the evolution of HPIV-3.

In summary, HPIVs, especially HPIV-3, were found to very important pathogens in hospitalized children with ARI in China. However, considering the limitations of this study, such as the populations sampled, the limitation of the diagnostic assays used, the timing and type of specimens collected, and specimen handling, the burden of HPIVs associated diseases maybe underestimated. This is the first report have described the phylogeny of HPIV-3 based on the complete HN sequence. Our results demonstrate phylogenetic classification based on the complete HN sequence provides the most rigorous method for molecular epidemiologic studies. These results highlight the importance of long-term epidemiology investigations of HPIVs for elucidation of herd immunity, genetic and antigenic mechanisms for better understanding of HPIVs infections.

## Materials and Methods

### Study Population

Nasopharyngeal aspirates (NPA) were collected from hospitalized children with ARI within 7 days of the onset of symptoms in Beijing Children’s hospital from May 2008 to September 2010. ARI was defined as the presence of constitutional signs or symptom of respiratory tract infection (i.e., cough, fast breathing, chestindrawing, etc.).The patients were diagnosed with upper respiratory tract infections, bronchitis, bronchiolitis and pneumonia. The clinical criteria for diagnosing pneumonia are the presence of lung infiltrates indicated by chest radiography [45]. Written informed consent for the use of their clinical samples was obtained from all guardians on the behalf of children involved in this study. This study was approved by the second session of the Ethics Review Committee of the Chinese Center for Disease Control and Prevention. For each participant, information on gender, age, clinical symptoms, and vaccination status was collected. All NPA specimens were stored at −70°C for further analysis.

### RNA Extraction

Viral genomic RNA was extracted from 140 µl of each NPA specimen, using the QIAamp viral RNA minikits (Qiagen, Shanghai, China) according to the manufacturer’s instructions. RNA pellets were resuspended in 60 µl of sterile distilled water and store at −70°C.

### Viral Detection

For HPIVs screening, one-step real-time reverse transcription polymerase chain reaction (RT-PCR) was performed using ABI 7500 real-time PCR system (Applied Biosystems, Foster City, CA). Briefly, amplification was carried out in 25 µl reaction volumes, including 5 µl of sample preparation and 20 µl of one-step RT-PCR master mix (One Step PrimeScriptTM RT-PCR Kit, TAKARA, Dalian, China). After a reverse transcription step at 42°C for 5 min followed by 10 sec of denaturation at 95°C, 40 cycles of two-step PCR was performed (5 sec at 95°C, 34 sec at 57°C). The primers and 6-carboxyfluorescein (FAM) labeled probes for detection of HPIV-3 were designed as previously described (Table 2) [46], [47]. Previously cultured material was used as a positive control and to validate the molecular assays.The real-time PCR procedures for detection of various viral gene including human respiratory syncytial virus (RSV) [48], human rhinovirus (HRVs) [49], adenovirus (AdV) [50], human coronaviruses (HCoVs: strains 229E, OC43, NL63 and HKU1) [51], [52] and human metapneumovirus (HMPV) [53]were conducted as previously described.

### RT-PCR Assays for HPIV-3

Samples that found positive for HPIV-3 by the real-time RT-PCR were tested by a classical RT-nested PCR to amplify the HN coding region in HPIV-3 gene. The outer primer set: 3HN+(5′-AAAAAGCACAGAACAGAAC-3′, nt 5379-nt 5397, sense) and 3HN- (5′-ACAGTGCCATTGTTAGATTCAG-3′, nt 8655-nt 8676, anti-sense) were used in a one-step RT-PCR. The first one-step RT-PCR assays (SuperScriptone-step RT-PCR with platinum Taq Kit, Invitrogen, USA) were undertaken in a 25 µl reaction volume containing 5 µl RNA-extract, 12.5 µl 2×reaction mix, 10 pmol of each primer, 0.5 µl of RT/Platinum Taq Mix, and RNase-free water to 25 µl. The reaction was carried with an initial reverse transcription step at 45°C for 30 min, followed by PCR activation at 94°C for 2 min, 30 cycles of amplification (15 sec at 94°C; 30 sec at 50°C; 2 min at 72°C) and final extension step at 72°C for 10 min. A nested PCR were carried out with the inner primer set 3HNn+(5′-ATGGAATACTGGAAGCACACCAACCAC-3′, nt 6806-nt 6832, sense) and 3HNn- (5′-TATCTCGAGTTATGATTAACTGCAGC-3′, nt 8514-nt 8539, antisense). The nested reaction mixture was composed of 1 unit of Taq polymerase, 1 µl of a 25 mMdNTP-mix, 2.5 mM MgSO_4_ 5 µl 10×PCR buffer (Platinum Taq DNA Polymerase High Fidelity Kit, Invitrogen, USA) and 10 pmol of forward and reverse primers in 50 µl reaction volume. As template 2 µl of the outer PCR product was added. The cycling conditions were as follows: an initial denaturation at 94°C for 2 min, followed by 30 cycles of amplification (94°C for 1 min, 50°C for 1 min and 72°C for 1 min), and a final extension of 5 min at 72°C. PCR products were analyzed by 1.5% agarose gel electrophoresis and purified using the QIAquick gel extraction kit (QIAGEN Inc., Chatsworth, CA). The purified PCR products were than sequenced with primers 3HNn+, P3−(5′-CTGAATTGTAAGAAGCCTTGT-3′, nt 7094-nt 7114, anti-sense), PF+(5′-CTCGAGGTTGTCAGGATATAG-3′, nt 7437-nt 7457, sense), P5+(5′-AACTGTGTTCAACTCCCAAAG-3′, nt 7605-nt 7625, sense) and P6+(5′-CAAGTTGGCATAGCAAGTTAC-3′, nt 8082-nt 8102, sense) using the ABI PRISM BigDye Terminator v3.0 Cycle Sequencing kit (Applied Biosystems, USA) on an ABI PRISM 3100 DNA sequencer (Applied Biosystems) according to the manufacturer’s instructions.

### Nucleotide Sequence and Phylogenetic Analysis

The sequences proof reading and editing was conducted with Sequencher™(Gene Codes Corporation). The obtained consensus sequences were compared with the prototype HPIV-3 sequences available in GenBank database using BLAST analysis (NCBI BLAST server). Nucleotide acid and deduced amino acid sequences of HPIV-3 were analyzed and aligned with BioEdit software (Tom Hall, North Carolina State University, Carolina, USA). Pairwise distances of nucleotide sequences in the 65 sequence alignment were calculated using Kimura 2-parametermethod within MEGA 5.0 package. The distance were sorted into three categories: Strain, the distance between strains within sub-clusters; Sub-cluster, the distances between strains across sub-cluster; and Cluster, the distance between strains across clusters, these data were used to generate histograms of the distribution of pairwise distance. The phylogenetic analyses were performed (MEGA version 5.0) by the neighbor-joining method using Kimura 2-parameters as substitution model, with statistical significance of phylogenies estimated by bootstrap analysis with 1,000 pseudoreplicate datasets. 42 representative nucleotide sequences were deposited in GenBank under accession numbers: GU732130-71.
